# Improved detection of influenza A virus from blue‐winged teals by sequencing directly from swab material

**DOI:** 10.1002/ece3.5232

**Published:** 2019-05-11

**Authors:** Lucas M. Ferreri, Lucia Ortiz, Ginger Geiger, Gonzalo P. Barriga, Rebecca Poulson, Ana Silvia Gonzalez‐Reiche, Jo Anne Crum, David Stallknecht, David Moran, Celia Cordon‐Rosales, Daniela Rajao, Daniel R. Perez

**Affiliations:** ^1^ Poultry Diagnostic and Research Center, Department of Population Health, College of Veterinary Medicine University of Georgia Athens Georgia; ^2^ Centro de Estudios en Salud Universidad del Valle de Guatemala Guatemala City Guatemala; ^3^ Laboratory of Emerging Viruses, Virology Program Institute of Biomedical Sciences, Faculty of Medicine Universidad de Chile Santiago Chile; ^4^ Southeastern Cooperative Wildlife Disease Study, Department of Population Health, College of Veterinary Medicine University of Georgia Athens Georgia; ^5^ Department of Genetics and Genomic Sciences Icahn School of Medicine at Mount Sinai New York New York

**Keywords:** blue‐winged teal, Guatemala, Illumina, influenza A virus, next‐generation sequencing, wild bird

## Abstract

**Abstract:**

The greatest diversity of influenza A virus (IAV) is found in wild aquatic birds of the orders Anseriformes and Charadriiformes. In these birds, IAV replication occurs mostly in the intestinal tract. Fecal, cloacal, and/or tracheal swabs are typically collected and tested by real‐time RT‐PCR (rRT‐PCR) and/or by virus isolation in embryonated chicken eggs in order to determine the presence of IAV. Virus isolation may impose bottlenecks that select variant populations that are different from those circulating in nature, and such bottlenecks may result in artifactual representation of subtype diversity and/or underrepresented mixed infections. The advent of next‐generation sequencing (NGS) technologies provides an opportunity to explore to what extent IAV subtype diversity is affected by virus isolation in eggs. In the present work, we evaluated the advantage of sequencing by NGS directly from swab material of IAV rRT‐PCR‐positive swabs collected during the 2013–14 surveillance season in Guatemala and compared to results from NGS after virus isolation. The results highlight the benefit of sequencing IAV genomes directly from swabs to better understand subtype diversity and detection of alternative amino acid motifs that could otherwise escape detection using traditional methods of virus isolation. In addition, NGS sequencing data from swabs revealed reduced presence of defective interfering particles compared to virus isolates. We propose an alternative workflow in which original swab samples positive for IAV by rRT‐PCR are first subjected to NGS before attempting viral isolation. This approach should speed the processing of samples and better capture natural IAV diversity.

**OPEN RESEARCH BADGES:**

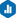

This article has earned an Open Data Badge for making publicly available the digitally‐shareable data necessary to reproduce the reported results. The data is available at https://doi.org/10.5061/dryad.3h2n106.

## 1 INTRODUCTION

Influenza A viruses (IAVs), in the family Orthomyxoviridae, carry a genome composed of eight negative‐sense RNA segments. Influenza A viruses are classified into subtypes based on the antigenic characteristics of the viral surface glycoproteins, hemagglutinin (HA), and neuraminidase (NA). Wild aquatic birds, especially birds in the orders Anseriformes (ducks and geese) and Charadriiformes (gulls and shorebirds), are considered the natural hosts for 16 HA (H1‐16) and 9 NA (N1‐9) subtypes. In these birds, IAVs replicate primarily in the gastrointestinal tract in the absence of overt signs of disease. Influenza A viruses are excreted in fecal material and are naturally perpetuated through fecal–oral transmission. The segmented genome allows IAVs to exchange gene segments, and thus, strains representing most HA/NA combinations are found in nature (Munster & Fouchier, 2009; Webster, Bean, Gorman, Chambers, & Kawaoka, 1992). Additional HA and NA subtypes have been identified in IAV associated with fruit bats (H17N10 and H18N11), but there is no evidence of exchange of genetic material between bat and avian IAVs (Tong et al., 2012, 2013).

For IAV surveillance in wild birds, fecal, cloacal, oropharyngeal, and/or tracheal swabs are tested by rRT‐PCR and/or by virus isolation in embryonated chicken eggs (ECE) and/or tissue‐cultured cells (TC) (Webster, Cox, & Stohr, 2005). Virus isolation (VI) strongly depends on the quality of the swab material and the presence of an adequate amount of infectious virus. This approach can impose selective bottlenecks that may either change the viral genome consensus sequence (Varble et al., 2014), allow competition between strains present in the original sample, lower the detection of mixed infections (Lindsay et al., 2013), and/or generate a bias in favor of HA and NA subtype combinations that are better fit for replication in ECE. In addition, VI strongly depends on the preservation of viable viral particles, and depending on the number of passages required, it may take up to 2 weeks to obtain a positive virus isolate (Webster et al., 2005). Previous studies have reported that rRT‐PCR is more sensitive than VI for detection of IAV (Gonzalez‐Reiche, Muller, Ortiz, Cordon‐Rosales, & Perez, 2016; Munster & Fouchier, 2009; Runstadler et al., 2007) but differences may reflect the ability to detect RNA from noninfectious IAV (Brown, Poulson, Carter, Lebarbenchon, & Stallknecht, 2013). The utility of next‐generation sequencing (NGS) and the ability to sequence directly from the original swab material for rapid IAV detection will produce more complete and accurate data reflective of naturally occurring subtypes and genetic diversity (McGinnis, Laplante, Shudt, & George, 2016; Zhao et al., 2016). In the present report, we show how performing NGS from original swab (swab‐NGS) material can improve IAV characterization from field samples.

## 2 MATERIALS AND METHODS

### 2.1 Sample collection, rRT‐PCR, and virus isolation (VI)

Samples were collected from hunter‐killed ducks during the winter migration season 2013–2014 in the villages of El Pumpo, in the department of Santa Rosa; Pasaco, in the department of Jutiapa and La Gomera, in the department of Escuintla, Guatemala. Sampling sites and tracheal and cloacal swab collection methods from birds followed previous descriptions (Gonzalez‐Reiche et al., 2012). Permits for sampling different bird species at sampling sites were obtained from the Center for Conservation Studies (CECON) and the National Council of Protected Areas (CONAP). Samples were preserved in 1 ml of virus transport medium (VTM, Medium 199 with Hanks balanced salt solution, 2 mM l‐glutamine, 0.5% bovine serum albumin, 0.35 g/L sodium bicarbonate, 2 × 10^6^ IU/L penicillin, 200 mg/L streptomycin, 2 × 10^6^ IU/L polymyxin B, 250 mg/L gentamycin, 0.5 × 10^6^ IU/L nystatin, 60 mg/L ofloxacin, and 0.2 g/L sulphamethoxazol) (Gonzalez‐Reiche et al., 2012) and stored at −70°C until used. All samples were tested for the presence of the IAV matrix (M) gene RNA by real‐time reverse‐transcriptase polymerase chain reaction (rRT‐PCR) (Spackman et al., 2002). Swabs showing Ct values <40 were considered positive. The details of the methods for RNA extraction, molecular testing, and virus isolation have been described elsewhere (Gonzalez‐Reiche et al., 2012). Viral isolation was attempted for rRT‐PCR IAV‐positive samples. Briefly, 200 µl of VTM from cloacal swab samples was inoculated into the allantoid cavity of 9‐day‐old specific pathogen‐free ECEs, and the eggs were then incubated for 72 hr and harvested in accordance with standardized protocols described in the WHO Manual on Animal Influenza Diagnosis and Surveillance (Webster et al., 2005). Collected allantoid fluids were tested by the hemagglutination assay to assess for presence of IAV as described (Webster et al., 2005). Briefly, allantoid fluid‐containing virus was serially diluted twofold using phosphate‐buffered saline (pH 7.4) and mixed 1:1 with 0.5% chicken red blood cells in V‐bottom 96‐well plates. Reading was performed after 45 min. Allantoid fluids that tested negative were diluted in half with phosphate‐buffered saline (pH 7.4) and inoculated in a new batch of ECE. The process was repeated up three times as needed.

### 2.2 Swab sample preparation, RNA extraction, and multisegment RT‐PCR (MS‐RT‐PCR)

Swabs embedded in a volume of 0.2–0.5 ml VTM were thawed on ice and vortexed for 1 min at room temperature. Samples were centrifuged for 3 min at 13,000 *g* to clarify the supernatant (Conceicao‐Neto et al., 2015). A 100 µl volume of either allantoid fluid or swab material was used for RNA extraction using the MagNA Pure LC RNA Isolation Kit—High Performance (Roche) on the MagNaPure LC instrument (Roche). The MS‐RT‐PCR was set up with minor modifications from a previously described method (Mena et al., 2016; Zhou et al., 2009). Briefly, 2.5 µl of RNA was used as template in a 25 µl MS‐RT‐PCR (Superscript III high‐fidelity RT‐PCR kit, ThermoFisher Co.); primer sequences and concentrations were as follows: Opti1‐F1 5′‐GTTACGCGCCAGCAAAAGCAGG‐3′ (0.06 µM); Opti1‐F2 5′‐GTTACGCGCCAGCGAAAGCAGG‐3′ (0.14 µM); Opti1‐R1 5′‐GTTACGCGCCAGTAGAAACAAGG‐3′ (0.2 µM). The cycling conditions were 55°C for 2 min, 42°C for 1 hr, 94°C for 2 min, 5 cycles (94°C for 30 s, 44°C for 30 s, 68°C for 3.5 min), followed by 26 cycles (94°C for 30 s, 55°C for 30 s, 68°C for 3.5 min) and a final extension of 68°C for 10 min. MS‐RT‐PCR products were analyzed in 0.8% agarose gel to confirm influenza genome amplification.

### 2.3 Amplicon purification and library preparation

Amplicons from MS‐RT‐PCRs were cleaned by 0.45× of Agencourt AMPure XP Magnetic Beads (Beckman Coulter) according to manufacturer's protocol and eluted in 30 µl of HyClone molecular biology water (Genesee Scientific). Concentration of the eluate was measured using the Qubit dsDNA HS Assay kit (ThermoFisher) on the Qubit 3.0 fluorometer (ThermoFisher). Amplicons were normalized to 0.2 ng/µl. Adapters were added by tagmentation using Nextera XT DNA library preparation kit (Illumina). The reaction was set up using 40% of the suggested final volume. Libraries were purified using 0.7× Agencourt AMPure XP Magnetic Beads, and fragment size distribution was analyzed on the Agilent Bioanalyzer using the High Sensitivity DNA kit (Agilent). Next, samples were normalized to 4 nM and pooled. The loading concentration of the pooled libraries was 15 pM. Libraries were sequenced using the MiSeq v2, 300 cycle reagent Kit (Illumina) in a paired‐end fashion (150 × 2).

### 2.4 Genome assembly and variant analysis

Genome assembly was performed using a pipeline previously developed at Icahn School of Medicine at Mount Sinai by Harm Van Bakel and described (Mena et al., 2016). Briefly, low‐quality sequences and adapters were removed by Cutadapt (Martin, 2011) from paired fastq files. An initial assembly was done using the inchworm component of Trinity (Grabherr et al., 2011) and viral contigs bearing internal deletions were identified by BLAT (Kent, 2002) mapping against nonredundant IRD reference sequences. Afterward, breakpoint‐spanning kmers from the assembly graph were removed by repeating the inchworm assembly, and the resulting contigs were then oriented and trimmed to remove low‐coverage ends and any extraneous sequences beyond the conserved IAV termini. To improve contiguity, the CAP3 (Huang & Madan, 1999) assembler was used and contigs from the same segment were merged if their ends overlapped by at least 25 nt. Finally, assembly contigs and contiguity were assessed for all segments by mapping sequence reads back to the final assembly using Burrows‐Wheeler Alignment (Li & Durbin, 2009). For the H5 HA segments, we also performed de novo assembly using Trinity (Haas et al., 2013). Pairwise alignment did not show any differences in the HA segment regardless of the assembly approach. Nucleotide variant analysis was performed using the program LoFreq (Wilm et al., 2012) following the Genome Analysis Toolkit best practices (Van der Auwera et al., 2013). The cutoff for minor variant analysis was arbitrarily set at 1,000× based on similar analysis found in the literature (Grubaugh et al., 2019; McCrone & Lauring, 2016; Wilm et al., 2012). Briefly, after removing adapters using Cutadapt, reads were mapped back to their reference using the option mem from BWA (Li & Durbin, 2009). Formatting the data for input to GATK was made using Picard (http://broadinstitute.github.io/picard/). Reads were realigned using RealignerTargetCreator and IndelRealigner from GATK. Finally, base's qualities were recalculated using BaseRecalibrator from GATK. The resulted bam file was used to perform variant calling analysis by LoFreq.

### 2.5 Plots

Data analyses were performed using GraphPad Prism software, version 8 (GraphPad Software Inc., San Diego, CA, US). Values for coverage plots were plotted using Microsoft Excel (version 16.18) (Microsoft, Redmond, WA, USA) and aesthetically modified using Inkscape v0.48.1 (https://inkscape.org).

### 2.6 Phylogenetic analyses

We prepared an avian influenza virus database with 350 genomes selected from IRD (https://www.fludb.org/), GISAID (https://www.gisaid.org), and NCBI (https://www.ncbi.nlm.nih.gov) databases. Alignments were performed by MUSCLE (http://www.drive5.com/muscle/) (Edgar, 2004). Phylogenetic trees were constructed with MEGA6 (http://www.megasoftware.net) and IQ‐TREE on the IQ‐TREE web server (http://www.cibiv.at/software/iqtree/) (Trifinopoulos, Nguyen, von Haeseler, & Minh, 2016) by using the maximum‐likelihood (ML) method. Robustness of tree topologies was assessed with 1,000 bootstrap replicates. Phylogenetic trees were constructed using ML inference with the general time‐reversible (GTR)+G (HA, NA, PB2, PB1, PA, NP, and NS) or GTR+G+I (NS) nucleotide substitution model.

## 3 RESULTS

### 3.1 Influenza A virus detection from field samples was increased by the swab‐NGS protocol

During the 2013–2014 IAV surveillance season in Guatemala, 579 paired tracheal/cloacal swab samples were obtained from wild aquatic birds, particularly blue‐winged teals (Table 1). Screening for the IAV matrix (M) gene by rRT‐PCR resulted in 74 IAV‐positive samples with Ct values ranging from 18.5 to 39.3 (prevalence of 12.8%). Positive samples were subsequently tested by H5 subtype‐specific rRT‐PCR (Spackman et al., 2002); nine samples (1.5% prevalence) yielded Ct values ranging from 28.1 to 36.6. This set of 74 IAV rRT‐PCR‐positive samples was further characterized by and compared under two NGS protocols. In the first, we followed a traditional protocol by attempting VI in ECE. Virus isolates were subsequently subjected to RNA extraction, followed by IAV genome amplification using multisegment RT‐PCR (MS‐RT‐PCR) and sequencing by NGS (VI‐NGS). In the second protocol, RNA was extracted directly from the original swab material, and the influenza genome was amplified by MS‐RT‐PCR and then sequenced by NGS (swab‐NGS).

**Table 1:** IAV‐positive samples and genomes obtained by swab‐NGS and VI‐NGS

Virus	Short name^a^	Genome assembly (missing segment)^b^	VI (# passages)^c^	HAU^d^
A/blue‐winged teal/Guatemala/CIP049‐I_H115‐29/2013 (H3N3)	115‐29_I	Complete	+ (1)	256
A/blue‐winged teal/Guatemala/CIP049‐S_H115‐29/2013 (H3N3)	115‐29_S	Complete	n/a	n/a
A/blue‐winged teal/Guatemala/CIP049‐H116‐05/2013 (H3,6N1,3)	116‐05	Complete	+ (2)	128
A/blue‐winged teal/Guatemala/CIP049‐H116‐07/2013 (H5N3)	116‐07	Complete	−	n/a
A/blue‐winged teal/Guatemala/CIP049‐H116‐08/2013 (H11N3)	116‐08	Incomplete (2, 3, 5, 8)	−	n/a
A/blue‐winged teal/Guatemala/CIP049‐H116‐10/2013 (H5N3)	116‐10	Complete	−	n/a
A/blue‐winged teal/Guatemala/CIP049‐H116‐120/2013 (H14N3)	116‐120	Complete	+ (3)	512
A/blue‐winged teal/Guatemala/CIP049‐I_H116‐16/2013 (H3N8)	116‐16_I	Complete	+ (1)	512
A/blue‐winged teal/Guatemala/CIP049‐S_H116‐16/2013 (H3N8)	116‐16_S	Incomplete (3, 4*, 6*)	n/a	n/a
A/blue‐winged teal/Guatemala/CIP049‐I_H116‐17/2013 (H3N2)	116‐17_I	Complete	+ (1)	2048
A/blue‐winged teal/Guatemala/CIP049‐S_H116‐17/2013 (H3N2)	116‐17_S	Complete	n/a	n/a
A/blue‐winged teal/Guatemala/CIP049‐H116‐22/2013 (H5N3)	116‐22	Complete	−	n/a
A/blue‐winged teal/Guatemala/CIP049‐H116‐26/2013 (H5Nx)	116‐26	Incomplete (1, 3, 6, 7, 8)	n/a	n/a
A/blue‐winged teal/Guatemala/CIP049‐I_H116‐48/2013 (H3,6N1,3)	116‐48_I	Complete	+ (2)	256
A/blue‐winged teal/Guatemala/CIP049‐S_H116‐48/2013 (H6N1,2)	116‐48_S	Complete	n/a	n/a
A/blue‐winged teal/Guatemala/CIP049‐H116‐50/2013 (H5N3)	116‐50	Complete	−	n/a
A/blue‐winged teal/Guatemala/CIP049‐H116‐51/2013 (H5N3)	116‐51	Complete	−	n/a
A/blue‐winged teal/Guatemala/CIP049‐I_H116‐76/2013 (H3N2,4)	116‐76_I	Complete	+ (1)	2048
A/blue‐winged teal/Guatemala/CIP049‐S_H116‐76/2013 (H3N2)	116‐76_S	Complete	n/a	n/a
A/blue‐winged teal/Guatemala/CIP049‐I_H116‐84/2013 (H6N2)	116‐84_I	Complete	+ (1)	1,024
A/blue‐winged teal/Guatemala/CIP049‐S_H116‐84/2013 (H6N2)	116‐84_S	Complete	n/a	n/a
A/blue‐winged teal/Guatemala/CIP049‐H116‐96/2013 (H5N2)	116‐96	Complete	−	n/a
A/blue‐winged teal/Guatemala/CIP049‐I_H116‐97/2013 (H4N8)	116‐97_I	Complete	+ (1)	256
A/blue‐winged teal/Guatemala/CIP049‐S_H116‐97/2013 (HxN8)	116‐97_S	Incomplete (2, 4)	n/a	n/a
A/blue‐winged teal/Guatemala/CIP049‐I_H117‐123/2013 (H14N3)	117‐123_I	Complete	+ (3)	128
A/blue‐winged teal/Guatemala/CIP049‐S_H117‐123/2013 (H14Nx)	117‐123_S	Incomplete (1, 2, 7, 6)	n/a	n/a
A/blue‐winged teal/Guatemala/CIP049‐H117‐125/2013 (H1N3)	117‐125	Complete	+ (1)	2048
A/blue‐winged teal/Guatemala/CIP049‐I_H117‐13/2013 (H14N3)	117‐13_I	Complete	+ (1)	512
A/blue‐winged teal/Guatemala/CIP049‐S_H117‐13/2013 (H14N3)	117‐13_S	Incomplete (2, 6*)	n/a	n/a
A/blue‐winged teal/Guatemala/CIP049‐H117‐130/2013 (H2N2)	117‐130	Complete	+ (2)	1,024
A/blue‐winged teal/Guatemala/CIP049‐I_H117‐143/2013 (H14N3)	117‐143_I	Complete	+ (1)	1,024
A/blue‐winged teal/Guatemala/CIP049‐S_H117‐143/2013 (HxNx)	117‐143_S	Incomplete (2, 4, 6, 8)	n/a	n/a
A/blue‐winged teal/Guatemala/CIP049‐H117‐34/2013 (H14N3)	117‐34	Complete	+ (1)	512
A/blue‐winged teal/Guatemala/CIP049‐H117‐36/2013 (H14N5)	117‐36	Complete	+ (1)	256
A/blue‐winged teal/Guatemala/CIP049‐H117‐38/2013 (H14N5)	117‐38	Complete	−	n/a
A/blue‐winged teal/Guatemala/CIP049‐H117‐42/2013 (H5N3)	117‐42	Complete	−	n/a
A/blue‐winged teal/Guatemala/CIP049‐H117‐99/2013 (H14N5)	117‐99	Complete	+ (2)	256
A/blue‐winged teal/Guatemala/CIP049‐H118‐23/2014 (H8N3,4)	118‐23	Complete	n/a	n/a
A/blue‐winged teal/Guatemala/CIP049‐I_H118‐64/2014 (H8N4)	118‐64_I	Complete	+ (2)	512
A/blue‐winged teal/Guatemala/CIP049‐S_H118‐64/2014 (H8N4)	118‐64_S	Complete	n/a	n/a
A/blue‐winged teal/Guatemala/CIP049‐H118‐81/2014 (H8N4)	118‐81	Complete	−	n/a
A/blue‐winged teal/Guatemala/CIP049‐H118‐82/2014 (HxNx)	118‐82	Incomplete (2, 4, 5, 6, 8)	−	n/a
A/blue‐winged teal/Guatemala/CIP049‐H119‐01/2014 (H12N3)	119‐01	Complete	−	n/a
A/blue‐winged teal/Guatemala/CIP049‐H119‐16/2014 (H12N4)	119‐16	Complete	−	n/a
A/blue‐winged teal/Guatemala/CIP049‐I_H120‐33/2014 (H12N4)	120‐33_I	Complete	+ (2)	512
A/blue‐winged teal/Guatemala/CIP049‐S_H120‐33/2014 (H12N4)	120‐33_S	Complete	n/a	n/a
A/blue‐winged teal/Guatemala/CIP049‐H121‐01/2014 (H12N7)	121‐01	Complete	−	n/a
A/blue‐winged teal/Guatemala/CIP049‐H121‐09/2014 (H7N7)	121‐09	Complete	+ (1)	32
A/blue‐winged teal/Guatemala/CIP049‐H121‐14/2014 (H7N7)	121‐14	Complete	+ (2)	16
A/blue‐winged teal/Guatemala/CIP049‐H121‐36/2014 (H2N9)	121‐36	Complete	−	n/a

a A unique identifier. Unique identifier with an “_I” and a “_S” indicates pair samples that produce NGS data by both VI‐NGS and swab‐NGS, respectively.

b Genome assembly is indicated by either complete or incomplete genomes. Gene segment number with missing sequence information is indicated in parentheses and correspond to 1 (PB2), 2 (PB1), 3 (PA), 4 (HA), 5 (NP), 6 (NA), 7 (M), and 8 (NS).

c VI, virus isolation. Number in parentheses indicate number of ECE passages that were required to produce a virus isolate. A (−) indicates a sample that was negative for virus isolation after three blind passages in ECE (but positive for swab‐NGS). A n/a corresponds to pair “_S” samples.

d Virus titers in ECE's allantoic fluid as measured by hemagglutination units (HAU) using chicken red blood cells.

**Figure 1:**
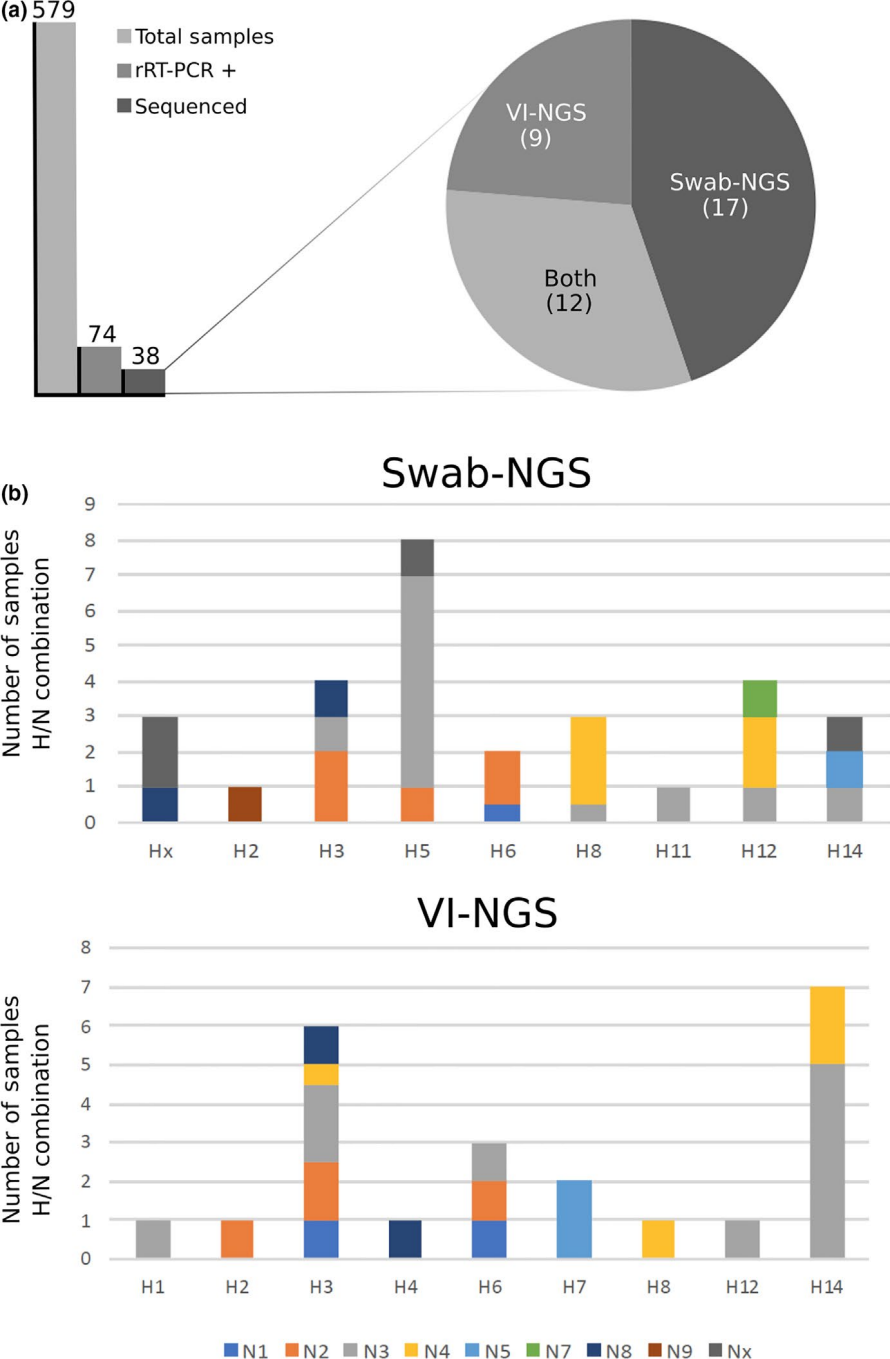
Characterization of the 2013–2014 IAV surveillance season using swab‐NGS and VI‐NGS. (a) 579 samples were collected of which 74 were positive by M gene segment rRT‐PCR. 38 samples resulted in virus sequence information by NGS of which nine full genome sequences were obtained only by VI‐NGS, 17 only by swab‐NGS and 12 from both protocols. (b) Subtype combination frequency in swab‐NGS and VI‐NGS is shown. The * indicates the most common HA subtype identified by swab‐NGS (H5) and VI‐NGS (H14), respectively

VI in ECEs resulted in 21 positive samples (28.4% of IAV rRT‐PCR‐positive samples). The number of passages needed for VI varied between 1 and 3 among samples (Table 1). All 21 virus isolates produced complete genome assemblies by NGS. The subtypes sequenced represented 14 different HA/NA combinations including two different mixed infections (Table 1 and Figure 1). None of the nine H5 IAV rRT‐PCR‐positive samples were isolated in eggs. The most common HA subtype obtained by VI was the H14.

By swab‐NGS, 29 samples were amplified by MS‐RT‐PCR (39.2% of IAV rRT‐PCR positives) and 21 of those produced complete genome assemblies, whereas eight had varying degrees of completeness (Table 1 and Figure 1). Swab‐NGS revealed 16 defined HA/NA combinations plus 3 that could not provide resolution of the HA and/or NA. Swab‐NGS led to the characterization of eight H5 subtype strains (H5N2, H5N3 [*n* = 6], and H5Nx) that were not identified by VI, including two that were initially negative by H5 rRT‐PCR. The rRT‐PCR utilizes a probe that targets the HA segment in the H5 subtype‐specific assay. No significant correlation between Ct values from H5‐specific rRT‐PCR and NGS coverage of the HA segment was observed (*R* ^2^ 0.002; *p* value 0.92). Except for the swab sample 116‐26, the rest of the H5 strains were completely sequenced and assembled. In addition, swab‐NGS resulted in eight subtype combinations that were not detected by VI: H2N9, H5N2, H5N3, H11N3, H12N3, H12N7 and mixed infections H6N(1/2) and H8N(4/3). Conversely, VI‐NGS resulted in five subtype combinations that were not captured by swab‐NGS including H1N3, H2N2, H7N7 and mixed infections H3N2/4, and H3/6N3/1. Of note, the H5 was the most prevalent subtype obtained by swab‐NGS. Taken together, the combination of swab‐NGS and VI‐NGS produced 21 different subtype combinations, of which eight would have been missed if we had relied only on VI‐NGS to characterize the 2013–2014 IAV season in wild birds in Guatemala. Phylogenetic analysis (available via datadryad.org) revealed that genomes obtained by either protocol belong to the North American lineage in agreement with most of the previously described isolates for this region (Gonzalez‐Reiche et al., 2012, 2016, 2017).

### 3.2 Molecular analysis shows markers of resistance to NA/M2 inhibitors, increased virulence in mammals, and two different PB1‐F2 ORFs

In order to better define the animal and public health risk of these viruses, molecular markers associated with drug resistance, enhanced transmission, and virulence to mammals were analyzed. No mammalian‐associated virulence markers in PB2 (E627K and D701N) (Hatta, Gao, Halfmann, & Kawaoka, 2001; Li et al., 2005; Shinya et al., 2004; Subbarao, London, & Murphy, 1993), PA (S409N) (Yamayoshi et al., 2014), or NS1 protein (T92E) (Ayllon & Garcia‐Sastre, 2015) were found for any of the samples. Sample 117‐123 (H14N3) contained markers for antiviral resistance on M2 (V27A and S31N) and NA (H274Y, oseltamivir resistance) along with the PB1‐F2 N66S marker, which is related to increased virulence in mammals (Kosik, Holly, & Russ, 2013; Krumbholz et al., 2011). Next‐generation sequencing data revealed that the PB1‐F2 open reading frame (ORF) was present in two different lengths of 87 and 90 amino acids both typically found in other IAVs from wild birds. There was no discernable relationship between the length of PB1‐F2 and the virus' ability to grow in ECE. Of note, all of the 87 amino acid‐long PB1‐F2 sequences (*n* = 14 samples) carried N66, whereas the 90 amino acid‐long counterparts carried S66 (19 samples), N66 (3 samples), or T66 (3 samples).

### 3.3 Swab‐NGS detects an unusual cleavage site motif for low‐pathogenicity H5 subtype viruses

Transition from low to high pathogenicity depends on the acquisition of several basic amino acids on the cleavage site of the HA protein. Subtypes that can naturally acquire this property have been so far restricted to H5 and H7. Protein sequence analyses of the different H5 (116‐96, 116‐07, 116‐22, 116‐50, 117‐42, and 116‐26) and H7 (121‐09 and 121‐14) subtypes revealed motifs consistent with low‐pathogenicity avian influenza virus strains (H5 motif ^P6^PQRETR*GLF and H7 motif ^P7^PENPKTR*GLF). Interestingly, we also identified 2 H5 HA sequences with the unusual motif ^P6^PQRE**P**R*GLF that has not been previously described for this subtype. The read coverage for the P2 site on these samples (116‐10 and 116‐51) was 7,220× and 5,752×, respectively. Variant analysis was carried out in order to assess the presence of variant populations at these nucleotide positions: No variants were detected suggesting a unique population for this molecular marker in these samples. The biological significance of such motif in nature and/or for in vitro or in ovo growth remains to be determined.

### 3.4 Comparison of VI‐NGS and swab‐NGS shows differences in nucleotide and amino acid consensus sequences

**Figure 2:**
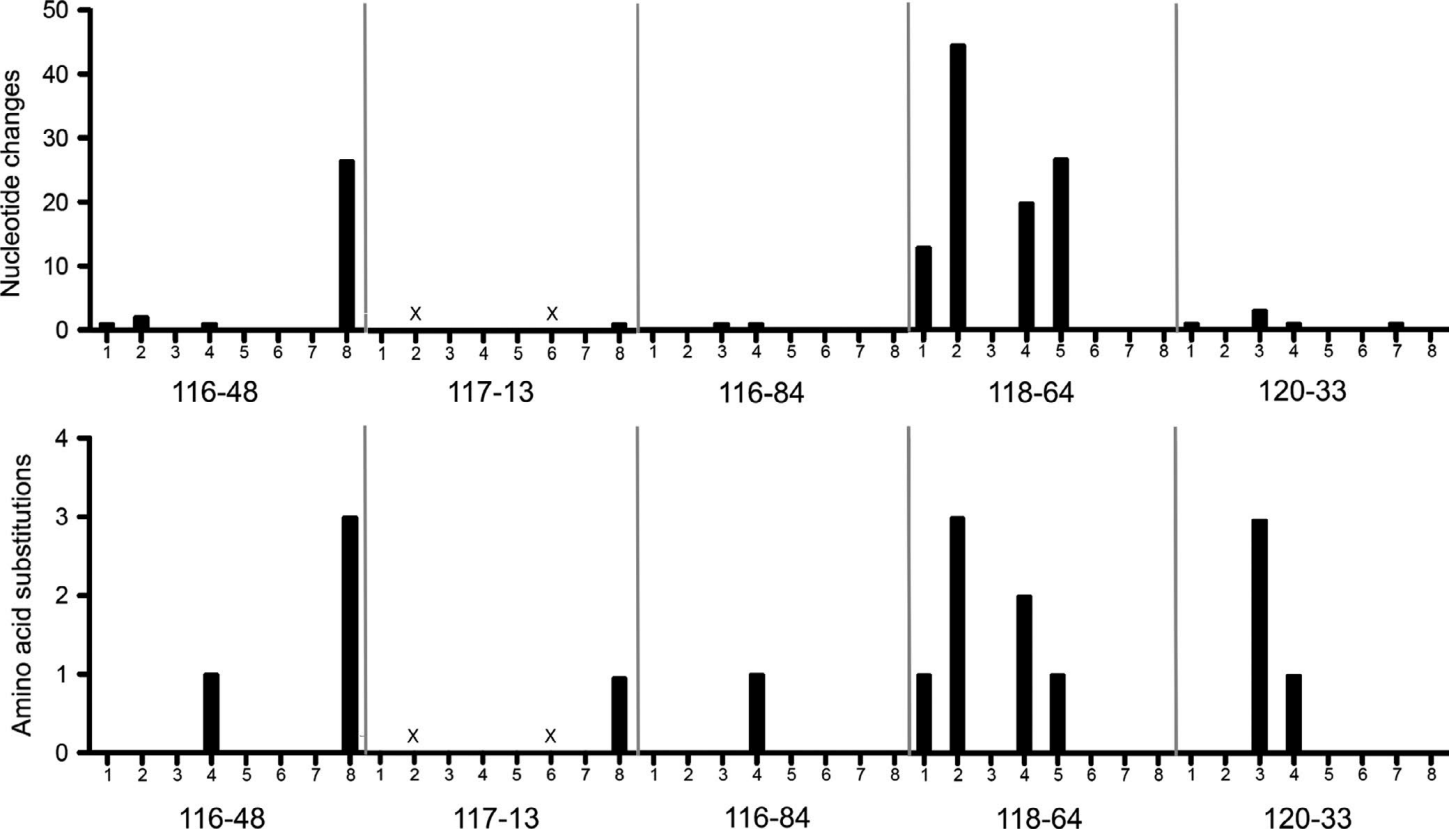
Nucleotide and amino acid changes in samples sequenced by swab‐NGS and VI‐NGS. Only samples that showed at least one amino acid discrepancy between the swab‐NGS and VI‐NGS protocols are shown. Numbers in the X‐axis correspond to gene segments as 1 (PB2), 2 (PB1), 3 (PA), 4 (HA), 5 (NP), 6 (NA), 7 (M) and 8 (NS). For sample 116‐48, segment 4 corresponds to H6 HA. Marked as “X” are gene segments whose sequence was not captured by swab‐NGS

Since modern IAV surveillance efforts have relied on sequencing of virus isolates, it has not been possible to ascertain the impact of ECEs (or tissue culture) on the selection of variants and/or subtype combinations. In this report, we sequenced 12 paired samples by swab‐NGS and VI‐NGS, which allowed evaluation of the consensus sequence variability between the virus in the original swab material and the ECE counterpart. Of these 12, 4 samples (115‐29, 116‐17, 116‐76, and 117‐143) showed no differences across their genome. In other paired samples, such as 116‐16, 116‐97, and 117‐123, only synonymous nucleotide differences were observed. Nonsynonymous differences were observed in paired samples 116‐84 (HA G122R), 117‐13 (NS1 A60V), and 120‐33 (PA L120I, V122A, and E154D; HA D243V) (Figure 2). Sample 116‐76 showed a H3N2 subtype by swab‐NGS but a mixed infection H3N2/4 by VI‐NGS; however, all shared segments showed 100% nt identity. Sample 116‐48 also exhibited discrepancies in the subtype characterization with a H6N1/2 mixed virus population by swab‐NGS and H3/6N1/3 (no N2) by VI‐NGS. In addition, the H6 HA of sample 116‐48 showed one amino acid difference between the paired samples (H6 HA L120F). Sample 116‐48 also contained 26‐nt differences in the NS segment, resulting predominately in synonymous changes with discrepancies in three amino acids in the NS1 ORF (NS1 T129I, N139D, and A197T). Interestingly, sample 118‐64 showed an unusual number of nucleotide differences in four gene segments: 13 in PB2, 45 in PB1, 20 in HA, and 27 in NP, leading to the following amino acid discrepancies: PB2 I147V; PB1 D177E, I368V and E383D; HA V41I and I354V; and NP S417N. Altogether, these results suggest that not all field samples are under selective pressure during VI in ECE.

### 3.5 Fixed amino acid substitutions after ECE were detected as minor variants by swab‐NGS pior isolation

The presence of minor variants helps the population to adapt to environmental changes. To assess the presence of minor variants that were later selected under VI, we carried out minor variant analysis. We used paired samples from swab‐NGS and VI‐NGS on sites where amino acid differences were detected. We analyzed samples 117‐13 (NS1), 116‐48 (HA and NS1), 116‐84 (HA), 118‐64 (PB2, PB1, HA and NP), and 120‐33 (PA and HA) (Table 2). Taken together, the swab‐NGS and VI‐NGS data revealed 17 amino acid differences due to the selection of alternative nucleotides after VI in ECE. Ten of those alternative nucleotides were detected as minor variants in the analysis of the swab‐NGS data with a frequency ≥0.1 (and <0.6), one with a frequency of 0.036 (in the PA segment of sample 120‐33 [PA c389]), whereas the remaining six samples showed minor variants below the limit of detection. Minor variants in the swab‐NGS became the dominant variants in the analysis of VI‐NGS. Further analysis of the VI‐NGS data revealed that minor variants were below limit of detection with the exceptions of minor variants in samples 117‐13 (NS1 c205) and 116‐84 (HA g381), which were present with a frequency of 0.458 and 0.077, respectively. Coincidentally, the minor variants in the samples 117‐13 and 116‐84 correspond to dominant variants in the swab‐NGS. Overall, these analyses further add to the notion of the selective pressure imposed on viruses during VI in ECE.

**Table 2:** Comparison of nucleotide variant analysis at sites of amino acid substitutions

Sample	Gene	Substitutions	Position	swab‐NGS				VI‐NGS			
				Reference	Variant	Depth	Frequency	Reference	Variant	Depth	Frequency
117‐13	NS1	A60V	205	C	T	6,093	0.193	T	C	10,170	0.458
116‐48	HA (H6)	L120F	375	C	*bld*	1,165	*bld*	T	*bld*	437^a^	*bld*
	NS1	T129I	412	C	T	5,252	0.106	T	*bld*	37,869	*bld*
		N139D	441	A	G	5,436	0.101	G	*bld*	35,820	*bld*
		A197T	615	G	A	5,120	0.196	A	*bld*	31,139	*bld*
116‐84	HA	G122R	381	G	*bld*	4,809	*bld*	A	G	11,034	0.077
118‐64	PB2	I147V	466	A	G	8,363	0.573	G	*bld*	136^a^	*bld*
	PB1	D177E	555	T	G	1,840	0.306	G	*bld*	4,896	*bld*
		I368V	1,126	A	G	2,232	0.320	G	*bld*	272^a^	*bld*
		E383D	1,173	G	T	1,765	0.324	T	*bld*	256^a^	*bld*
	HA	V41I	140	G	*bld*	4,059	*bld*	A	*bld*	497^a^	*bld*
		I354V	1,079	A	*bld*	4,495	*bld*	G	*bld*	1521	*bld*
	NP	S417N	1,295	G	A	6,219	0.546	A	*bld*	3,407	*bld*
120‐33	PA	L120I	382	C	A	5,279	0.388	A	*bld*	27,095	*bld*
		V122A	389	T	C	4,768	0.036	C	*bld*	26,044	*bld*
		E154D	486	A	*bld*	4,875	*bld*	T	*bld*	13,265	*bld*
	HA	D243V	745	A	*bld*	8,197	*bld*	T	*bld*	24,814	*bld*

Note

Minor nucleotide variant analysis was performed using samples that showed amino acid differences between the swab‐NGS and VI‐NGS protocols.

Reference, nucleotide in the reference sequence. Variant, variant nucleotide observed. Depth, depth of coverage of the corresponding segment. Frequency, frequency of the variant nucleotide in the corresponding segment. *bld*, below limit of detection (frequency >0.002).

a Corresponds to sites with depth <1,000×. The cutoff for minor variant analysis was arbitrarily set at 1,000× based on similar analysis found in the literature (Grubaugh et al., 2019; McCrone & Lauring, 2016; Wilm et al., 2012).

### 3.6 Swab‐NGS allows for assessment of genome integrity in the natural host

**Figure 3:**
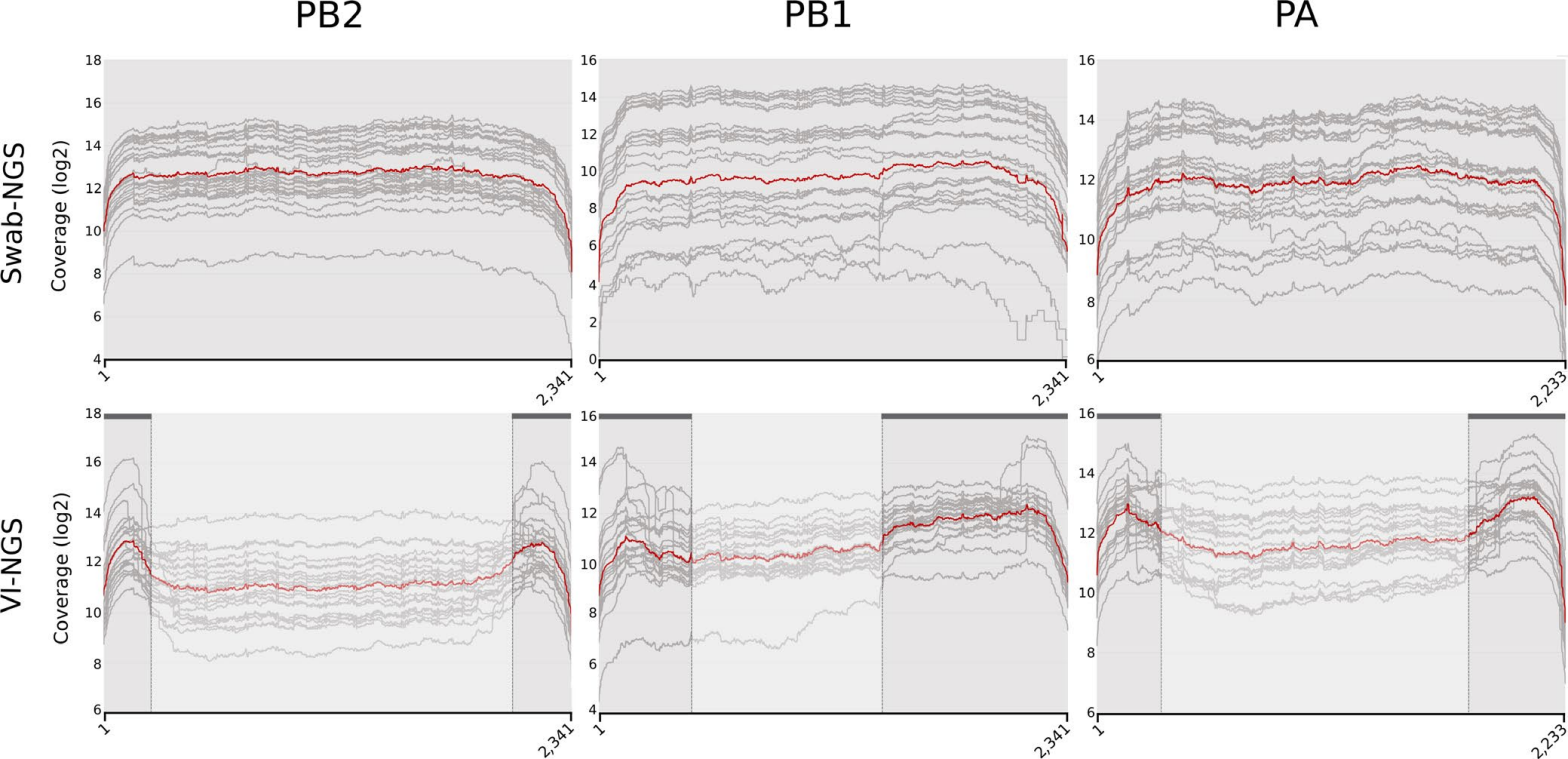
Coverage plot of polymerase segments from swab‐NGS compared to VI‐NGS. Gray lines show the coverage distribution from each individual sample. The red line depicts the geometric mean. Dark gray horizontal lines and vertical dashed lines in VI‐NGS show the DIPs' approximate breakpoints. Clouded areas indicate relatively lower sequence coverage compared to the 5′ and 3′ ends of the segment. First and last nucleotide positions are shown in the X‐axis

Genome integrity in IAV viruses may be disrupted by the generation of defective interfering particles (DIP) (Von Magnus, 1954). These particles are produced when viruses are passaged at high multiplicity of infection (MOI), leading to truncated forms of one or more gene segments, typically the polymerase gene segments but carrying intact packaging signals (Davis, Hiti, & Nayak, 1980; Nayak, 1980; Nayak, Chambers, & Akkina, 1985; Odagiri & Tashiro, 1997). Previously, DIPs were detected in influenza viruses obtained from naturally infected human and chicken samples (Chambers & Webster, 1987; Saira et al., 2013). Within NGS data, DIPs are usually inferred by the formation a valley‐like shape in the coverage distribution plot, produced by a bias of reads toward the 3′ and 5′ ends of the gene segments (Lee, Lee, Tang, Loh, & Koay, 2016; Saira et al., 2013). We compared the coverage distribution of PB2, PB1, and PA produced by swab‐NGS and VI‐NGS. The mean coverage for each segment was calculated and depicted in order to highlight the topography of the coverage plots (Figure 3). As it is easily observed, swab‐NGS revealed no indication of major DIP populations in the samples. In contrast, VI‐NGS showed reads compatible with the presence of DIPs in the sample. These observations suggest that DIPs observed from VI‐NGS may not reflect the production of such particles in nature.

## 4 DISCUSSION

Historically, IAV detection in wild bird samples has been performed by virus isolation in ECE with or without prior testing of the samples by classical RT‐PCR or rRT‐PCR. The entire process is time‐consuming and occasionally requires multiple ECE passages before a virus is isolated. Our group has been performing IAV surveillance in wild birds in Guatemala and Argentina for more than a decade (Gonzalez‐Reiche et al., 2012, 2016, 2017; Pereda et al., 2008; Rimondi et al., 2018, 2011; Xu et al., 2012). In both of these countries, we have described the circulation of highly diverse IAVs in terms of subtype combinations as well as phylogenetic characteristics. Due to multiple limitations including reliable access to specific pathogen‐free ECEs in other countries, increasing importation costs, and regulatory restrictions that prevent rapid sample evaluation, we sought to improve virus characterization by performing NGS directly from swab samples. Although NGS has been previously used to characterize IAV from original samples (Ren et al., 2013; Seong et al., 2016; Wang et al., 2008; Zhao et al., 2016), such studies were not aimed at comparing it to viral isolation or determining whether it could result in different consensus sequences. Using swabs collected from wild birds in Guatemala during the 2013–2014 season, we amplified and obtained sequence information by swab‐NGS from 29 out of 74 IAV rRT‐PCR‐positive samples. By comparison, 21 samples (out of 74) resulted in virus isolates with only 12 in common between the two approaches. Even though total numbers favored swab‐NGS, the subtype landscape depicted by both protocols showed substantial differences. Surveillance activities from previous seasons in Guatemala using VI have shown the H14 as the most prevalent subtype (Gonzalez‐Reiche et al., 2016). Using VI‐NGS, the same scenario was depicted for season 2013–2014. However, detection by swab‐NGS shifted the subtype landscape to H5 (*n* = 8) as the most prevalent subtype of the season. In the previous five seasons, a total of seven isolates were subtyped as H5 highlighting the improvement given by swab‐NGS (Gonzalez‐Reiche et al., 2012, 2016, 2017). In this study, there was no significant correlation between the Ct values of the H5‐specific rRT‐PCR and the swab‐NGS coverage for the H5 HA. However, further studies are warranted as perhaps correlations could be established if samples were processed for both rRT‐PCR and swab‐NGS as soon as collected. Two of the H5 HA sequences showed molecular markers in the cleavage site that have not been previously described. The coverage was high, and no variants were found for this site in independent samples from two different birds. Since this cleavage motif has not been described before, it remains to be determined whether it would affect virus isolation in ECE.

Discrepancies in consensus sequences may arise after VI in vitro or in ovo. Fixation of these variants may be the product of selection resulting in increased fitness for replication in an artificial substrate. Minor variants were detected by swab‐NGS from four viruses (117‐13, 116‐48, 118‐64, and 120‐33) that became dominant variants after VI on ECE. At these same positions, VI‐NGS data revealed that, with the exception of 117‐13 (NS1 c205) and 116‐84 (HA g381), minor variants were below limit of detection, indicating fixation of these amino acids. Further studies beyond the scope of this report would be needed to elucidate the biological significance of these substitutions. Although not all swab/VI paired samples had sequence discrepancies, the restriction of some viruses to be isolated in addition to amino acids substitutions present in others underlines the stringency imposed by genetic bottlenecks. We also found discrepancies in the resolution of mixed infections, which is in agreement with previous reports (Lindsay et al., 2013; Wang et al., 2008). Competition between subtypes with variable fitness for ECE isolation may account for the discrepancies observed between swab‐NGS and VI‐NGS with respect to mixed infections (Stallknecht et al., 2012; Varich, Gitelman, Shilov, Smirnov, & Kaverin, 2008).

At least one virus field sample (117‐123) showed molecular markers of antiviral resistance to adamantane and oseltamivir, stressing natural circulation of drug‐resistant IAV strains in wild aquatic birds. This sample also contained the PB1‐F2 S66 marker, which has been associated with increased pathogenicity in mammals (Conenello et al., 2011). All sequenced PB1 segments showed either the 87 or 90 amino acid‐long PB1‐F2 protein sequence, which is common in isolates from avian species (James et al., 2016). We also found N and T at position 66; interestingly, PB1‐F2 T66 was only found in samples (*n* = 3 samples) sequenced through swab‐NGS. The presence of PB1‐F2 T66 in databases is low, suggesting either low circulation of this marker in avian species and/or detection bias. In chickens, PB1‐F2 has been implicated in increasing the duration of virus shedding upon infection and lowering pathogenicity (Kamal et al., 2015; Krumbholz et al., 2011; Zell et al., 2007). The consequence of the total length and residue preference at position 66 in PB1‐F2 for fitness in natural hosts remains to be determined.

The VI‐NGS coverage topography suggested the presence of DIPs in the samples (Lee et al., 2016; Saira et al., 2013). Since the viral load in the original swab material is unknown, DIPs may have emerged due to large quantities of replicating fit virus particles/viral segments in the egg inoculum or swab samples used for isolation. Consistently, the original swab samples did not show the typical DIP topography in the coverage plots by swab‐NGS suggesting low prevalence of such particles in the wild. We must note, however, that the limited number of samples and species (all samples came from *Anas discors*) in this study precludes further speculation about the existence of DIPs in natural hosts.

**Figure 4:**
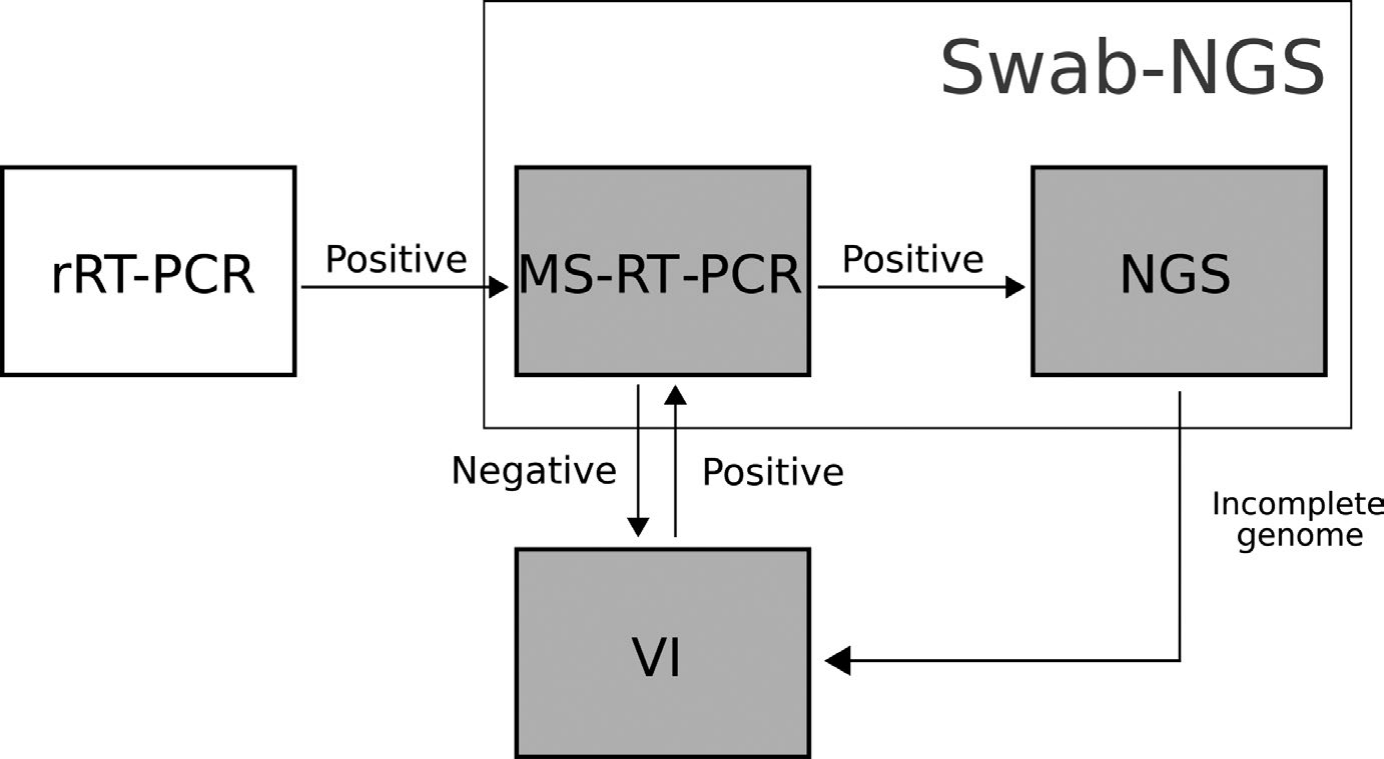
Proposed workflow. The alternative workflow proposes an initial screening by rRT‐PCR to identify IAV‐positive samples which are then subjected to MS‐RT‐PCR and NGS sequencing. Swab‐NGS samples with incomplete genomes and/or positive rRT‐PCR that failed the MS‐RT‐PCR procedure are processed for virus isolation either in ECE or TC. Virus isolates are eventually run by NGS

In summary, the combination of swab‐NGS and VI‐NGS led to more complete characterization of subtype diversity of IAVs in wild bird samples from Guatemala during the 2013–2014 season. The swab‐NGS protocol in the present work was applied on samples that were 3 years old and had more than one freeze–thaw cycle; thus, the integrity of the vRNA may have been affected. We are tempted to argue that the swab‐NGS would yield better results if applied on freshly collected samples. Thus, we propose a workflow (Figure 4) in which swab material is initially screened for positives by rRT‐PCR and then sequenced by swab‐NGS before attempting VI. Alternatively, samples could be subjected directly to swab‐NGS. It must be noted that if NGS capabilities are not readily available, MS‐RT‐PCR‐positive samples can be safely stored until NGS can be performed elsewhere. The IAV rRT‐PCR‐positive samples that fail the MS‐RT‐PCR protocol can then be processed for VI. Likewise, swab‐NGS samples that result in incomplete genomes could be completed with VI‐NGS. The swab‐NGS workflow is ultimately less time‐consuming and cost‐effective. The downside is that it does not allow for a more complete phenotypic characterization of the virus. If such characterizations are pursued, swab‐NGS and VI‐NGS represent complementary approaches since, as shown in the present work, they provide clues regarding selection of variants as well the genomic plasticity of the virus isolates. It is important to note that swab‐NGS may provide full virus genome information even from samples no longer viable for VI. In this case, synthetic DNA technology and reverse genetics offer the possibility to rescue a virus isolate from genomic information.

The swab‐NGS proposed in this report has already been successfully used with human samples (Meinel et al., 2018). In our hands, it has been successfully applied on field samples from a variety of species such as *A. discors*, *Anas platyrhynchos*, *Anas georgica*, *Netta peposaca*, and *Amazonetta brasiliensis* as well as swine samples (data not shown). Hence, the swab‐NGS workflow can be successfully applied to a variety of species. Newer sequencing technologies, such as nanopore sequencing (Keller et al., 2018a, 2018b), and protocol refinements could result in improvements of this workflow in order to speed up virus genomic characterization from original swab samples.

## CONFLICT OF INTEREST

None declared.

## AUTHORS' CONTRIBUTIONS

LMF designed studies, developed methods, analyzed data, and wrote and edited the manuscript. LO, DM, ASGR, and CCR collected and processed field samples, and edited the manuscript. GPB and ASGR performed phylogenetic analysis, RP, JAC, and DS analyzed data and edited the manuscript. DR edited the manuscript. DRP designed studies, analyzed data, and wrote and edited the manuscript.

## Data Availability

All sequences are publicly available in GenBank under accession numbers MK326645–MK326849 and MK327614–MK327786. Trees are publicly available at datadryad.org (https://doi.org/10.5061/dryad.3h2n106).

## References

[ece35232-bib-0001] Ayllon, J. , & Garcia‐Sastre, A. (2015). The NS1 protein: A multitasking virulence factor. Current Topics in Microbiology and Immunology, 386, 73–107. 10.1007/82_2014_400 25007846

[ece35232-bib-0002] Brown, J. D. , Poulson, R. , Carter, D. L. , Lebarbenchon, C. , & Stallknecht, D. E. (2013). Infectivity of avian influenza virus‐positive field samples for mallards: What do our diagnostic results mean? Journal of Wildlife Diseases, 49(1), 180–185. 10.7589/2011-11-322 23307386 PMC11373666

[ece35232-bib-0003] Chambers, T. M. , & Webster, R. G. (1987). Defective interfering virus associated with A/Chicken/Pennsylvania/83 influenza virus. Journal of Virology, 61(5), 1517–1523. 3573146 10.1128/jvi.61.5.1517-1523.1987 PMC254130

[ece35232-bib-0004] Conceicao‐Neto, N. , Zeller, M. , Lefrere, H. , De Bruyn, P. , Beller, L. , Deboutte, W. , … Matthijnssens, J. (2015). Modular approach to customise sample preparation procedures for viral metagenomics: A reproducible protocol for virome analysis. Scientific Reports, 5, 16532. 10.1038/srep16532 26559140 PMC4642273

[ece35232-bib-0005] Conenello, G. M. , Tisoncik, J. R. , Rosenzweig, E. , Varga, Z. T. , Palese, P. , & Katze, M. G. (2011). A single N66S mutation in the PB1‐F2 protein of influenza A virus increases virulence by inhibiting the early interferon response in vivo. Journal of Virology, 85(2), 652–662. 10.1128/JVI.01987-10 21084483 PMC3020033

[ece35232-bib-0006] Davis, A. R. , Hiti, A. L. , & Nayak, D. P. (1980). Influenza defective interfering viral RNA is formed by internal deletion of genomic RNA. Proceedings of the National Academy of Sciences of the United States of America, 77(1), 215–219. 6928614 10.1073/pnas.77.1.215 PMC348239

[ece35232-bib-0007] Edgar, R. C. (2004). MUSCLE: Multiple sequence alignment with high accuracy and high throughput. Nucleic Acids Research, 32(5), 1792–1797. 10.1093/nar/gkh340 15034147 PMC390337

[ece35232-bib-0008] Gonzalez‐Reiche, A. S. , Morales‐Betoulle, M. E. , Alvarez, D. , Betoulle, J. L. , Muller, M. L. , Sosa, S. M. , & Perez, D. R. (2012). Influenza A viruses from wild birds in Guatemala belong to the North American lineage. PLoS ONE, 7(3), e32873. 10.1371/journal.pone.0032873 22427902 PMC3302778

[ece35232-bib-0009] Gonzalez‐Reiche, A. S. , Muller, M. L. , Ortiz, L. , Cordon‐Rosales, C. , & Perez, D. R. (2016). Prevalence and diversity of low pathogenicity avian influenza viruses in wild birds in Guatemala, 2010–2013. Avian Diseases, 60(1 Suppl), 359–364. 10.1637/11130-050715-Reg 27309080 PMC5003613

[ece35232-bib-0010] Gonzalez‐Reiche, A. S. , Nelson, M. I. , Angel, M. , Muller, M. L. , Ortiz, L. , Dutta, J. , … Perez, D. R. (2017). Evidence of intercontinental spread and uncommon variants of low‐pathogenicity avian influenza viruses in ducks overwintering in Guatemala. mSphere, 2(2), e00362‐16. 10.1128/mSphere.00362-16 28405632 PMC5381266

[ece35232-bib-0011] Grabherr, M. G. , Haas, B. J. , Yassour, M. , Levin, J. Z. , Thompson, D. A. , Amit, I. , … Regev, A. (2011). Full‐length transcriptome assembly from RNA‐Seq data without a reference genome. Nature Biotechnology, 29(7), 644–652. 10.1038/nbt.1883 PMC3571712 21572440

[ece35232-bib-0012] Grubaugh, N. D. , Gangavarapu, K. , Quick, J. , Matteson, N. L. , De Jesus, J. G. , Main, B. J. , … Andersen, K. G. (2019). An amplicon‐based sequencing framework for accurately measuring intrahost virus diversity using PrimalSeq and iVar. Genome Biology, 20(1), 8. 10.1186/s13059-018-1618-7 30621750 PMC6325816

[ece35232-bib-0013] Haas, B. J. , Papanicolaou, A. , Yassour, M. , Grabherr, M. , Blood, P. D. , Bowden, J. , … Regev, A. (2013). De novo transcript sequence reconstruction from RNA‐seq using the Trinity platform for reference generation and analysis. Nature Protocols, 8(8), 1494–1512. 10.1038/nprot.2013.084 23845962 PMC3875132

[ece35232-bib-0014] Hatta, M. , Gao, P. , Halfmann, P. , & Kawaoka, Y. (2001). Molecular basis for high virulence of Hong Kong H5N1 influenza A viruses. Science, 293(5536), 1840–1842. 11546875 10.1126/science.1062882

[ece35232-bib-0015] Huang, X. , & Madan, A. (1999). CAP3: A DNA sequence assembly program. Genome Research, 9(9), 868–877. 10508846 10.1101/gr.9.9.868 PMC310812

[ece35232-bib-0016] James, J. , Howard, W. , Iqbal, M. , Nair, V. K. , Barclay, W. S. , & Shelton, H. (2016). Influenza A virus PB1‐F2 protein prolongs viral shedding in chickens lengthening the transmission window. Journal of General Virology, 97(10), 2516–2527. 10.1099/jgv.0.000584 27558742 PMC5078828

[ece35232-bib-0017] Kamal, R. P. , Kumar, A. , Davis, C. T. , Tzeng, W. P. , Nguyen, T. , Donis, R. O. , … York, I. A. (2015). Emergence of highly pathogenic avian influenza A(H5N1) virus PB1‐F2 variants and their virulence in BALB/c mice. Journal of Virology, 89(11), 5835–5846. 10.1128/JVI.03137-14 25787281 PMC4442455

[ece35232-bib-0018] Keller, M. W. , Rambo‐Martin, B. L. , Wilson, M. M. , Ridenour, C. A. , Shepard, S. S. , Stark, T. J. , … Barnes, J. R. (2018a). Author correction: Direct RNA sequencing of the coding complete influenza A virus genome. Scientific Reports, 8(1), 15746. 10.1038/s41598-018-34067-6 30341398 PMC6195582

[ece35232-bib-0019] Keller, M. W. , Rambo‐Martin, B. L. , Wilson, M. M. , Ridenour, C. A. , Shepard, S. S. , Stark, T. J. , … Barnes, J. R. (2018b). Direct RNA sequencing of the coding complete influenza A virus genome. Scientific Reports, 8(1), 14408. 10.1038/s41598-018-32615-8 30258076 PMC6158192

[ece35232-bib-0020] Kent, W. J. (2002). BLAT–the BLAST‐like alignment tool. Genome Research, 12(4), 656–664. 10.1101/gr.229202 11932250 PMC187518

[ece35232-bib-0021] Kosik, I. , Holly, J. , & Russ, G. (2013). PB1‐F2 expedition from the whole protein through the domain to aa residue function. Acta Virologica, 57(2), 138–148. 23600872 10.4149/av_2013_02_138

[ece35232-bib-0022] Krumbholz, A. , Philipps, A. , Oehring, H. , Schwarzer, K. , Eitner, A. , Wutzler, P. , & Zell, R. (2011). Current knowledge on PB1‐F2 of influenza A viruses. Medical Microbiology and Immunology, 200(2), 69–75. 10.1007/s00430-010-0176-8 20953627

[ece35232-bib-0023] Lee, H. K. , Lee, C. K. , Tang, J. W. , Loh, T. P. , & Koay, E. S. (2016). Contamination‐controlled high‐throughput whole genome sequencing for influenza A viruses using the MiSeq sequencer. Scientific Reports, 6, 33318. 10.1038/srep33318 27624998 PMC5022032

[ece35232-bib-0024] Li, H. , & Durbin, R. (2009). Fast and accurate short read alignment with Burrows‐Wheeler transform. Bioinformatics, 25(14), 1754–1760. 10.1093/bioinformatics/btp324 19451168 PMC2705234

[ece35232-bib-0026] Li, Z. , Chen, H. , Jiao, P. , Deng, G. , Tian, G. , Li, Y. , … Yu, K. (2005). Molecular basis of replication of duck H5N1 influenza viruses in a mammalian mouse model. Journal of Virology, 79(18), 12058–12064. 10.1128/JVI.79.18.12058-12064.2005 16140781 PMC1212590

[ece35232-bib-0027] Lindsay, L. L. , Kelly, T. R. , Plancarte, M. , Schobel, S. , Lin, X. , Dugan, V. G. , … Boyce, W. M. (2013). Avian influenza: Mixed infections and missing viruses. Viruses, 5(8), 1964–1977. 10.3390/v5081964 23921843 PMC3761236

[ece35232-bib-0028] Martin, M. (2011). Cutadapt removes adapter sequences from high‐throughput sequencing reads. Embnet Journal, 17, 10–12.

[ece35232-bib-0029] McCrone, J. T. , & Lauring, A. S. (2016). Measurements of intrahost viral diversity are extremely sensitive to systematic errors in variant calling. Journal of Virology, 90(15), 6884–6895. 10.1128/JVI.00667-16 27194763 PMC4944299

[ece35232-bib-0030] McGinnis, J. , Laplante, J. , Shudt, M. , & George, K. S. (2016). Next generation sequencing for whole genome analysis and surveillance of influenza A viruses. Journal of Clinical Virology, 79, 44–50. 10.1016/j.jcv.2016.03.005 27085509

[ece35232-bib-0031] Meinel, D. M. , Heinzinger, S. , Eberle, U. , Ackermann, N. , Schonberger, K. , & Sing, A. (2018). Whole genome sequencing identifies influenza A H3N2 transmission and offers superior resolution to classical typing methods. Infection, 46(1), 69–76. 10.1007/s15010-017-1091-3 29086356

[ece35232-bib-0032] Mena, I. , Nelson, M. I. , Quezada‐Monroy, F. , Dutta, J. , Cortes‐Fernandez, R. , Lara‐Puente, J. H. , … Garcia‐Sastre, A. (2016). Origins of the 2009 H1N1 influenza pandemic in swine in Mexico. Elife, 5, 10.7554/eLife.16777 PMC4957980 27350259

[ece35232-bib-0033] Munster, V. J. , & Fouchier, R. A. (2009). Avian influenza virus: Of virus and bird ecology. Vaccine, 27(45), 6340–6344. 10.1016/j.vaccine.2009.02.082 19840670

[ece35232-bib-0034] Nayak, D. P. (1980). Defective interfering influenza viruses. Annual Review of Microbiology, 34, 619–644. 10.1146/annurev.mi.34.100180.003155 7002033

[ece35232-bib-0035] Nayak, D. P. , Chambers, T. M. , & Akkina, R. K. (1985). Defective‐interfering (DI) RNAs of influenza viruses: Origin, structure, expression, and interference. Current Topics in Microbiology and Immunology, 114, 103–151. 3888540 10.1007/978-3-642-70227-3_3

[ece35232-bib-0036] Odagiri, T. , & Tashiro, M. (1997). Segment‐specific noncoding sequences of the influenza virus genome RNA are involved in the specific competition between defective interfering RNA and its progenitor RNA segment at the virion assembly step. Journal of Virology, 71(3), 2138–2145. 9032347 10.1128/jvi.71.3.2138-2145.1997 PMC191316

[ece35232-bib-0037] Pereda, A. J. , Uhart, M. , Perez, A. A. , Zaccagnini, M. E. , La Sala, L. , Decarre, J. , … Perez, D. R. (2008). Avian influenza virus isolated in wild waterfowl in Argentina: Evidence of a potentially unique phylogenetic lineage in South America. Virology, 378(2), 363–370. 10.1016/j.virol.2008.06.010 18632129 PMC2570041

[ece35232-bib-0038] Ren, X. , Yang, F. , Hu, Y. , Zhang, T. , Liu, L. , Dong, J. , … Jin, Q. (2013). Full genome of influenza A (H7N9) virus derived by direct sequencing without culture. Emerging Infectious Diseases, 19(11), 1881–1884. 10.3201/eid1911.130664 24206919 PMC3837655

[ece35232-bib-0039] Rimondi, A. , Gonzalez‐Reiche, A. S. , Olivera, V. S. , Decarre, J. , Castresana, G. J. , Romano, M. , … Perez, D. R. (2018). Evidence of a fixed internal gene constellation in influenza A viruses isolated from wild birds in Argentina (2006–2016). Emerging Microbes and Infections, 7(1), 194. 10.1038/s41426-018-0190-2 30482896 PMC6258671

[ece35232-bib-0040] Rimondi, A. , Xu, K. , Craig, M. I. , Shao, H. , Ferreyra, H. , Rago, M. V. , … Pereda, A. (2011). Phylogenetic analysis of H6 influenza viruses isolated from rosy‐billed pochards (*Netta peposaca*) in Argentina reveals the presence of different HA gene clusters. Journal of Virology, 85(24), 13354–13362. 10.1128/JVI.05946-11 21976652 PMC3233172

[ece35232-bib-0041] Runstadler, J. A. , Happ, G. M. , Slemons, R. D. , Sheng, Z. M. , Gundlach, N. , Petrula, M. , … Taubenberger, J. K. (2007). Using RRT‐PCR analysis and virus isolation to determine the prevalence of avian influenza virus infections in ducks at Minto Flats State Game Refuge, Alaska, during August 2005. Archives of Virology, 152(10), 1901–1910. 10.1007/s00705-007-0994-1 17541700 PMC2538573

[ece35232-bib-0042] Saira, K. , Lin, X. , DePasse, J. V. , Halpin, R. , Twaddle, A. , Stockwell, T. … INSIGHT FLU003 Study Group (2013). Sequence analysis of in vivo defective interfering‐like RNA of influenza A H1N1 pandemic virus. Journal of Virology, 87(14), 8064–8074. 10.1128/JVI.00240-13 23678180 PMC3700204

[ece35232-bib-0043] Seong, M. W. , Cho, S. I. , Park, H. , Seo, S. H. , Lee, S. J. , Kim, E. C. , & Park, S. S. (2016). Genotyping influenza virus by next‐generation deep sequencing in clinical specimens. Annals of Laboratory Medicine, 36(3), 255–258. 10.3343/alm.2016.36.3.255 26915615 PMC4773267

[ece35232-bib-0044] Shinya, K. , Hamm, S. , Hatta, M. , Ito, H. , Ito, T. , & Kawaoka, Y. (2004). PB2 amino acid at position 627 affects replicative efficiency, but not cell tropism, of Hong Kong H5N1 influenza A viruses in mice. Virology, 320(2), 258–266. 10.1016/j.virol.2003.11.030 15016548

[ece35232-bib-0045] Spackman, E. , Senne, D. A. , Myers, T. J. , Bulaga, L. L. , Garber, L. P. , Perdue, M. L. , … Suarez, D. L. (2002). Development of a real‐time reverse transcriptase PCR assay for type A influenza virus and the avian H5 and H7 hemagglutinin subtypes. Journal of Clinical Microbiology, 40(9), 3256–3260. 12202562 10.1128/JCM.40.9.3256-3260.2002 PMC130722

[ece35232-bib-0046] Stallknecht, D. E. , Luttrell, M. P. , Poulson, R. , Goekjian, V. , Niles, L. , Dey, A. , … Webster, R. G. (2012). Detection of avian influenza viruses from shorebirds: Evaluation of surveillance and testing approaches. Journal of Wildlife Diseases, 48(2), 382–393. 10.7589/0090-3558-48.2.382 22493113 PMC3584701

[ece35232-bib-0047] Subbarao, E. K. , London, W. , & Murphy, B. R. (1993). A single amino acid in the PB2 gene of influenza A virus is a determinant of host range. Journal of Virology, 67(4), 1761–1764. 8445709 10.1128/jvi.67.4.1761-1764.1993 PMC240216

[ece35232-bib-0048] Tong, S. , Li, Y. , Rivailler, P. , Conrardy, C. , Castillo, D. A. , Chen, L. M. , … Donis, R. O. (2012). A distinct lineage of influenza A virus from bats. Proceedings of the National Academy of Sciences of the United States of America, 109(11), 4269–4274. 10.1073/pnas.1116200109 22371588 PMC3306675

[ece35232-bib-0049] Tong, S. , Zhu, X. , Li, Y. , Shi, M. , Zhang, J. , Bourgeois, M. , … Donis, R. O. (2013). New world bats harbor diverse influenza A viruses. PLoS Path, 9(10), e1003657. 10.1371/journal.ppat.1003657 PMC3794996 24130481

[ece35232-bib-0050] Trifinopoulos, J. , Nguyen, L. T. , von Haeseler, A. , & Minh, B. Q. (2016). W‐IQ‐TREE: A fast online phylogenetic tool for maximum likelihood analysis. Nucleic Acids Research, 44(W1), W232–W235. 10.1093/nar/gkw256 27084950 PMC4987875

[ece35232-bib-0051] Van der Auwera, G. A. , Carneiro, M. O. , Hartl, C. , Poplin, R. , Del Angel, G. , Levy‐Moonshine, A. , DePristo, M. A. (2013). From FastQ data to high confidence variant calls: The Genome Analysis Toolkit best practices pipeline. Current Protocols in Bioinformatics, 43, 11.10.1–11.10.33. 10.1002/0471250953.bi1110s43 PMC4243306 25431634

[ece35232-bib-0052] Varble, A. , Albrecht, R. A. , Backes, S. , Crumiller, M. , Bouvier, N. M. , Sachs, D. , … tenOever, B. R. (2014). Influenza A virus transmission bottlenecks are defined by infection route and recipient host. Cell Host and Microbe, 16(5), 691–700. 10.1016/j.chom.2014.09.020 25456074 PMC4272616

[ece35232-bib-0053] Varich, N. L. , Gitelman, A. K. , Shilov, A. A. , Smirnov, Y. A. , & Kaverin, N. V. (2008). Deviation from the random distribution pattern of influenza A virus gene segments in reassortants produced under non‐selective conditions. Archives of Virology, 153(6), 1149–1154. 10.1007/s00705-008-0070-5 18414973

[ece35232-bib-0054] Von Magnus, P. (1954). Incomplete forms of influenza virus. Advances in Virus Research, 2, 59–79. 13228257 10.1016/s0065-3527(08)60529-1

[ece35232-bib-0055] Wang, R. , Soll, L. , Dugan, V. , Runstadler, J. , Happ, G. , Slemons, R. D. , & Taubenberger, J. K. (2008). Examining the hemagglutinin subtype diversity among wild duck‐origin influenza A viruses using ethanol‐fixed cloacal swabs and a novel RT‐PCR method. Virology, 375(1), 182–189. 10.1016/j.virol.2008.01.041 18308356 PMC2397557

[ece35232-bib-0056] Webster, R. G. , Bean, W. J. , Gorman, O. T. , Chambers, T. M. , & Kawaoka, Y. (1992). Evolution and ecology of influenza A viruses. Microbiological Reviews, 56(1), 152–179. 1579108 10.1128/mr.56.1.152-179.1992 PMC372859

[ece35232-bib-0057] Webster, R. , Cox, N. , & Stohr, K. (2005). WHO manual on animal influenza diagnosis and surveillance. Retrieved from http://www.who.int/csr/resources/publications/influenza/whocdscsrncs20025rev.pdf

[ece35232-bib-0058] Wilm, A. , Aw, P. P. , Bertrand, D. , Yeo, G. H. , Ong, S. H. , Wong, C. H. , … Nagarajan, N. (2012). LoFreq: A sequence‐quality aware, ultra‐sensitive variant caller for uncovering cell‐population heterogeneity from high‐throughput sequencing datasets. Nucleic Acids Research, 40(22), 11189–11201. 10.1093/nar/gks918 23066108 PMC3526318

[ece35232-bib-0059] Xu, K. , Ferreri, L. , Rimondi, A. , Olivera, V. , Romano, M. , Ferreyra, H. , … Perez, D. R. (2012). Isolation and characterization of an H9N2 influenza virus isolated in Argentina. Virus Research, 168(1–2), 41–47. 10.1016/j.virusres.2012.06.010 22709552 PMC5003612

[ece35232-bib-0060] Yamayoshi, S. , Yamada, S. , Fukuyama, S. , Murakami, S. , Zhao, D. , Uraki, R. , … Kawaoka, Y. (2014). Virulence‐affecting amino acid changes in the PA protein of H7N9 influenza A viruses. Journal of Virology, 88(6), 3127–3134. 10.1128/JVI.03155-13 24371069 PMC3957961

[ece35232-bib-0061] Zell, R. , Krumbholz, A. , Eitner, A. , Krieg, R. , Halbhuber, K. J. , & Wutzler, P. (2007). Prevalence of PB1‐F2 of influenza A viruses. Journal of General Virology, 88(Pt 2), 536–546. 10.1099/vir.0.82378-0 17251572

[ece35232-bib-0062] Zhao, J. , Liu, J. , Vemula, S. V. , Lin, C. , Tan, J. , Ragupathy, V. , … Hewlett, I. (2016). Sensitive detection and simultaneous discrimination of influenza A and B viruses in nasopharyngeal swabs in a single assay using next‐generation sequencing‐based diagnostics. PLoS ONE, 11(9), e0163175. 10.1371/journal.pone.0163175 27658193 PMC5033603

[ece35232-bib-0063] Zhou, B. , Donnelly, M. E. , Scholes, D. T. , St George, K. , Hatta, M. , Kawaoka, Y. , & Wentworth, D. E. (2009). Single‐reaction genomic amplification accelerates sequencing and vaccine production for classical and Swine origin human influenza A viruses. Journal of Virology, 83(19), 10309–10313. 10.1128/JVI.01109-09 19605485 PMC2748056

